# Amphetamines signal through intracellular TAAR1 receptors coupled to Gα_13_ and Gα_S_ in discrete subcellular domains

**DOI:** 10.1038/s41380-019-0469-2

**Published:** 2019-08-09

**Authors:** Suzanne M. Underhill, Patrick D. Hullihen, Jingshan Chen, Cristina Fenollar-Ferrer, M. A. Rizzo, Susan L. Ingram, Susan G. Amara

**Affiliations:** 1 National Institute of Mental Health, National Institutes of Health, Bethesda, MD 20892 USA; 2 Department of Physiology, University of Maryland School of Medicine, Baltimore, MD 21201 USA; 3 Department of Neurological Surgery, Oregon Health & Sciences University, Portland, OR 97239 USA

**Keywords:** Neuroscience, Cell biology

## Abstract

The extensive use of amphetamines to treat attention deficit hyperactivity disorders in children provides a compelling rationale for understanding the mechanisms of action of amphetamines and amphetamine-related drugs. We have previously shown that acute amphetamine (AMPH) regulates the trafficking of both dopamine and glutamate transporters in dopamine neurons by increasing activation of the small GTPase RhoA and of protein kinase A. Here we demonstrate that these downstream signaling events depend upon the direct activation of a trace amine-associated receptor, TAAR1, an intracellular G-protein coupled receptor (GPCR) that can be activated by amphetamines, trace amines, and biogenic amine metabolites. Using cell lines and mouse lines in which TAAR1 expression has been disrupted, we demonstrate that TAAR1 mediates the effects of AMPH on both RhoA and cAMP signaling. Inhibition of different Gα signaling pathways in cell lines and in vivo using small cell-permeable peptides confirms that the endogenous intracellular TAAR1 couples to G_13_ and to G_S_ α-subunits to increase RhoA and PKA activity, respectively. Results from experiments with RhoA- and PKA-FRET sensors targeted to different subcellular compartments indicate that AMPH-elicited PKA activation occurs throughout the cell, whereas G_13_-mediated RhoA activation is concentrated near the endoplasmic reticulum. These observations define TAAR1 as an obligate intracellular target for amphetamines in dopamine neurons and support a model in which distinct pools of TAAR1 mediate the activation of signaling pathways in different compartments to regulate excitatory and dopaminergic neurotransmission.

## Introduction

Amphetamine (AMPH) and a variety of AMPH analogues have well-established actions as substrates and competitive inhibitors of the dopamine, norepinephrine, and/or serotonin transporters (DAT, NET, and/or SERT). These compounds gain entry into neurons through DAT, NET, and/or SERT, displace neurotransmitters from vesicular stores and facilitate efflux through the plasma membrane carriers. In addition, we have shown that AMPH activates signal transduction pathways that lead to the internalization of the DAT and of the neuronal glutamate transporter, EAAT3 [1, 2], through a series of intracellular events that appear to be distinct from the established actions of competitive inhibitors of dopamine (DA) transport. Application of AMPH to cell lines, cultured DA neurons or midbrain slices, leads to the activation of the small GTPases, RhoA and Rac-1. Activation of RhoA and subsequent cytoskeletal remodeling mediate internalization of the transporters by an endocytic pathway. However, AMPH also modulates cAMP concentrations which stimulate protein kinase A (PKA)-phosphorylation of Rho, reducing its activity.

Cocaine and other drugs that inhibit the transport of AMPH readily block the effects of AMPHs on transporter trafficking [1], GTPase signaling, and PKA activation [2, 3] consistent with the idea that AMPHs trigger these events within the cell. The activation of both RhoA and PKA signaling pathways occurs in cells that lack the capacity for DA synthesis and vesicular packaging, which suggests that the effects of AMPH are not mediated by DA. These apparent direct effects of AMPH on signaling suggests the existence of a novel intracellular target for the drug and led us to examine potential intracellular targets for AMPH and other related compounds.

Here we show that the trace amine associated receptor 1 (TAAR1) serves as a direct intracellular target for amphetamines in DA neurons. TAAR1 is an intracellular GPCR stimulated by a variety of trace amines and monoamines, including AMPH [4, 5]. Multiple reports support a role for TAAR1 in the actions of psychostimulants. TAAR1 knockout (KO) mice are hypersensitive to AMPH [6], and antagonists of TAAR1 modulate the locomotor activation produced by methamphetamine [7], cocaine [8], and other psychostimulants. The addictive properties of methamphetamine [9, 10] and cocaine [11–13] also appear to be regulated by the receptor.

Using transgenic mice and cell lines that lack the TAAR1 receptor we now demonstrate that the intracellular effects of  AMPH, including both the elevation in cAMP and the increased RhoA activity, depend upon TAAR1 activation. We use a combination of gene disruption technologies, expression of targeted FRET sensors and the introduction of competitive peptides to prevent specific GPCR G-protein α-subunit interactions to determine the origin of and mechanism through which TAAR1 initiates both GTPase and PKA signaling cascades. We observe in cell lines and neurons that when TAAR1 is activated by AMPH within the cell, it couples through a G-protein α subunit, G_13_, commonly associated with RhoA GTPase activation. RhoA signaling is concentrated near the endoplasmic reticulum (ER). The intracellular AMPH-mediated activation of PKA by TAAR1 does not localize specifically to ER but appears more broadly distributed throughout the cell with most robust activation occurring within membranes not associated with lipid rafts.

## Materials and methods

Cell lines: HEK293 and SK-N-SH cells were obtained from ATTC and maintained in DMEM with 5% fetal bovine serum and Penn/Strep antibiotics. Cells were transfected with Lipofectamine 2000 according to the manufacturer’s directions (Invitrogen). The mutant G alpha- KO cell lines were generous gifts from Dr. Asuka Inoue (Graduate School of Pharmaceutical Sciences Tohoku University 6–3, Aoba, Aramaki, Aoba-ku, Sendai, Miyagi, 980–8578 Japan) and Dr. Jurgen Wess (NIDDK, NIH). Rescue of the G_12_ and G_13_ subunits was performed with plasmids graciously provided to us by Dr. David Sibley. TAAR1 gene KO in the HEK293 cell line was developed using CRISPR/Cas9 genome editing technology. The CRISPR guide RNA target site (CCTGTACAGTTTAATGGTGCTCA) near the 5′ end of the human TAAR1 gene was selected using the online tool at MIT from Dr. Feng Zhang’s laboratory (http://crispr.mit.edu). Oligonucleotides (AAACGTACAGTTTAATGGTGCTCAC and CATGTCAAATTACCACGAGTGCCAC) for the target were synthesized, annealed, and cloned into the CRISPR vector, SpCas9-2A-Puro (PX459, Addgene plasmid # 62988)[14], and used to generate a plasmid, named pCR3-hTA1, that encodes a CRISPR guide RNA (gRNA) against human TAAR1 gene. The pCR3-TA1 plasmid was introduced into HEK293 cells by transfection with Lipofectamine 2000 and cells stably expressing the vector were selected with puromycin. The human *TAAR1* gene in the stable cell lines was amplified by PCR with specific primers (hTA1F: GATTGACAGCCCTCAGGAATGATG and hTA1B: GAACTCAATTCCAAAAATAATTTACA) and screened for mutations with Surveyor Mutation Detection Kit (Transgenomic Limited). The human *TAAR1* gene PCR products with mutations were cloned into pCR2.1-TOPO vector (Thermo Fisher Scientific) and sequenced. A mutant HEK293 cell line with a deletion mutation of two nucleotides at the 27th tyrosine codon of human *TAAR1* gene in both alleles resulted in the formation of a premature stop codon, TAC (Tyr)- >TAG (Stop), which leads to the homozygous KO of the human *TAAR1* gene was selected. The mutant cell line was designated as *TAAR1* KO HEK293 cell line. To rescue the deficits due to the homozygous KO of the taar1 gene, we developed a mutant *TAAR1* gene that encodes wild-type (WT) TAAR1 protein but carries two point mutations at the guide RNA target site (C**T**CTGTA**T**AGTTTAATGGTGCTCA), which confers the resistance against the gRNA to the *TAAR1* gene.

*TAAR1* KO animals: The TAAR1 KO mice (Taar1 Ko mice) were a generous gift from Dr. Greg Miller from the NERC, NIH grant OD011103. Tissue derived from both male and female animals aged 12–24 weeks was used for the RhoA activation assays, as well as the biotinylation studies. Results were similar across the sexes and have been presented cumulatively here. Animals were housed with 12:12 h light/dark cycles and food/water was available ad libitum.

All assays were done in accordance with the National Institutes of Health Animal Care Committee or the OHSU Institutional Animal Care Committee.

^3^H-neurotransmitter uptake assays: DAT and EAAT3 function were assessed with ^3^H-neurotransmitter uptake assays. In all, 20 μM cold and 100 nM hot DA or 250 μM cold and 50 nM hot glutamate were used for 10 min in PBS supplemented with Mg^2+^ and Ca^2+^. In this, as with all cell culture assays, the procedure was performed from transfection to uptake at least three independent times. A single well is considered *n* for statistical purposes.

Immunochemistry: Cells were fixed with 4% paraformaldehyde for 20 min at 4 °C, permeabilized with 0.125% Triton X-100 in PBS and blocked with 10% normal goat serum (NGS) for 1 h. Primary antibodies were applied overnight in 5% NGS at 1:1000. Secondary antibodies were applied for 1–2 h at room temperature, diluted 1:1000 in 5% NGS. DAPI (1:10,000) was used to counterstain the nuclei for 5 min prior to mounting in Fluoromount Aquesous Mounting Medium (Sigma, F4680).

Confocal microscopy and FRET imaging: All imaging was performed on a Nikon A1Rsi confocal microscope. For FRET microcopy, cells were transfected and imaged 12–48 h later. In these assays, serum starved cells did not exhibit a remarkable difference from those maintained under normal conditions and the data have been pooled. Under 405 nm illumination, CFP and YFP images were collected. After background subtraction, CFP/YFP ratios were determined and normalized to the 2 min prior to treatment. Cells that did not respond to epinephrine for AKAR4 or calpeptin (Cytoskeleton Rho activator I) for the RhoA sensor were eliminated from analysis and included <~15% of the total cells.

Rho activation assays: Glutathione-S-transferase conjugated to Rho-binding domain of Rhotekin (GST–RBP) protein was grown in BL21 bacteria and isolated with glutathione-agarose, as described previously [15]. Cells or acute brain slices were treated and the cells were lysed in 1% Triton X-100, 150 mM NaCl, 5 mM EDTA, and 50 mM Tris, pH 7.5, containing a protease inhibitor. Cell lysates and GST–RBP beads were incubated overnight at 4 °C. Activated GTP-bound RhoA adhered to the GST–RBP. RhoA was eluted in loading buffer, run on Bis-Tris glycine gels and probed for with an antibody against RhoA. When these assays were performed on acute slices, similar to biotinylation assays, at least three slices were used on three different experimental days.

Biotinylation assays: Cell surface expression of proteins was assessed by biotinylation assays in cell culture and acute brain slices as described previously [1]. Briefly, after cells or tissue was treated with vehicle or experimental conditions, they were chilled to halt trafficking. Proteins located at the cell surface were biotinylated with 2 mg/ml sulfosuccinimidyl 2-(biotinamido) methyl-1,3-dithiopropionate (sulfo-NHS-SS-biotin) (Pierce, Rockford, IL). Labeling was quenched with 100 mM glycine and lysates were made of the preparation in 1% Triton X-100, 150 mM NaCl, 5 mM EDTA, and 50 mM Tris, pH 7.5, containing a protease inhibitor mixture (Roche Molecular Biochemicals, Indianapolis, IN). Biotinylated proteins were isolated by Ultralink immobilized NeutrAvidin beads (Pierce) and analyzed by western blot on Invitrogen Tris-Glycine gels.

Plasmid constructs: The small targeting motifs added by the Zhang laboratory to AKAR4 (Addgene #61619) and, here, to the Rho-FRET sensor are outlined in table 1. Briefly, the lipid raft targeting motif was derived from the Lyn kinase amino-terminus and the nonraft plasma membrane signaling was distinguished by a motif (CAAX) derived from the GTP-ase KRAS [16]. Cytosolic targeting was achieved by a carboxy-terminus (C-terminus) motif of a nuclear export signal [17] and targeting of AKAR4 to the ER was mediated by an amino-terminus domain derived from cytochrome 450 [18]. Motifs were added to the plasmids with NEB Q5 Site-Directed mutagenesis system.

**Table 1:** Targeting motifs for biosensors

Name	Targeting	Derived from	Terminus	Sequence	AKAR4 Addgene
NES	Cytosolic targeting	Nuclear export signal	C	EFLPPLERLTL	#64727
KRAS	Non-raft plasmid membrane	KRAS GTPase (V-Ki-ras2 Kirsten rat sarcoma viral oncogene homolog)	C	KKKKKKSKTKCVIM	#61621
ER	Endoplasmic reticulum	Cytochrome P450	N	MDPVVVLGLCLSCLLLLSLWKQSYGGGDP	#64733
Lyn	Lipid raft targeting	Lyn kinase (Lck/Yes novel tyrosine kinase)	N	MGCIKSKRKDKDP	#61620

The GPCR α-subunit interfering sequences were graciously provided by the Hamm lab at Vanderbilt University. The TAT peptides used in this study were designed based on their previous reports [19] as outlined in table 2, and ordered from LifeTein.

**Table 2:** Sequences for interfering with the α-subunits of GPCRs

α- subunit	Sequence
G_q_	MGLQLNLKEYNAV
G_11_	MGLQLNLKEYNLV
G_12_	MGLQENLKDIMLQ
G_13_	MGLHDNLKQLMLQ
G_S_	MGQRMHLRQYELL
Scrambled control	MGNGIKCLFNDKL

Electrophysiological recordings: Mice were deeply anesthetized with isoflurane and brains were rapidly removed and placed in ice cold “cutting buffer” containing (in mM): 75 NaCl, 2.5 KCl, 0.1 CaCl_2_, 6 MgSO_4_, 1.2 NaH_2_PO_4_, 25 NaHCO_3_, 2.5 D-Dextrose, and 50 Sucrose. Midbrain horizontal slices (~230  μm) were cut in 95% O_2_ and 5% CO_2_ oxygenated cutting buffer. Slices were incubated in warm (35 °C) 95% O_2_ and 5% CO_2_ oxygenated artificial cerebrospinal fluid (aCSF) containing kynurenic acid (500 nM) for at least 40 min and maintained at room temperature afterward until transfer to a recording chamber. The aCSF contained (in mM): 126 NaCl, 2.5 KCl, 2.4 CaCl_2_, 1.2 MgCl_2_, 1.2 NaH_2_PO_4_, 21.4 NaHCO_3_, 11.1 D-Dextrose, pH 7.4, and the osmolarity was adjusted to 300–310 mOsm. Whole cell patch clamp recordings were made from visually identified substantia nigra pars compacta neurons and further characterized by the presence of a hyperpolarization-activated membrane rectification (Ih current). Patch pipettes were pulled from borosilicate glass (1.5 mm diameter; WPI, Sarasota, FL) on a two-stage puller (PP83, Narishige, Tokyo, Japan). Pipettes had a resistance of 2–4 Mohm, when filled with the intracellular solution containing (in mM): 130 CsCl, 5.4 KCl, 0.1 CaCl_2_, 2MgCl_2_, 10 HEPES, 1.1 EGTA, 30 D-Dextrose, 4 Mg-ATP, and 1 Na-GTP; pH was adjusted to 7.2–7.3, and the osmolarity was adjusted to 280–290 mOsm. Whole cell series resistance was compensated by ~80%. Oxygenated aCSF at 32 °C was used for recording and extracellular solution exchange was achieved by height-controlled perfusion system with speed ~3–4 ml/min. Evoked excitatory synaptic currents were recorded at a holding potential of −70 mV. Currents were collected at 2 kHz and digitized at 5 kHz using either an Axopatch 700 A or 200B amplifiers controlled by Axograph Data Acquisition software. NMDAR EPSCs were evoked in nominally Mg^2+^-free aCSF solution containing 10 µM NBQX and 10 µM glycine with a bipolar stimulating electrode placed ~200–300 mm distally from the recorded cell. Stimulation pulses (0.02 ms) were delivered at 0.05 Hz. During each experiment, a voltage step of −10 mV from the holding potential was applied periodically to monitor cell capacitance and access resistance. Recordings in which access resistance or capacitance changed by >15% during the experiment were excluded from data analysis. To avoid activation of DA receptors and alpha-adrenergic receptors caused by increases in extracellular DA release, the following inhibitors were included in the aCSF recording solution: DA-D1: SCH 23390 1 µM; DA-D2: eticlopride 10 µM; alpha-adrenergic receptors: prazosin 1 µM. Bicuculline (10 µM) and strychnine (1 µM) were also included in the bath during recordings to eliminate GABAA and glycine receptor activation. The G_13_ inhibitor and scrambled peptides were included in the intracellular pipette solutions. One neuron was recorded per slice and two slices were typically used per mouse.

**Fig. 1:**
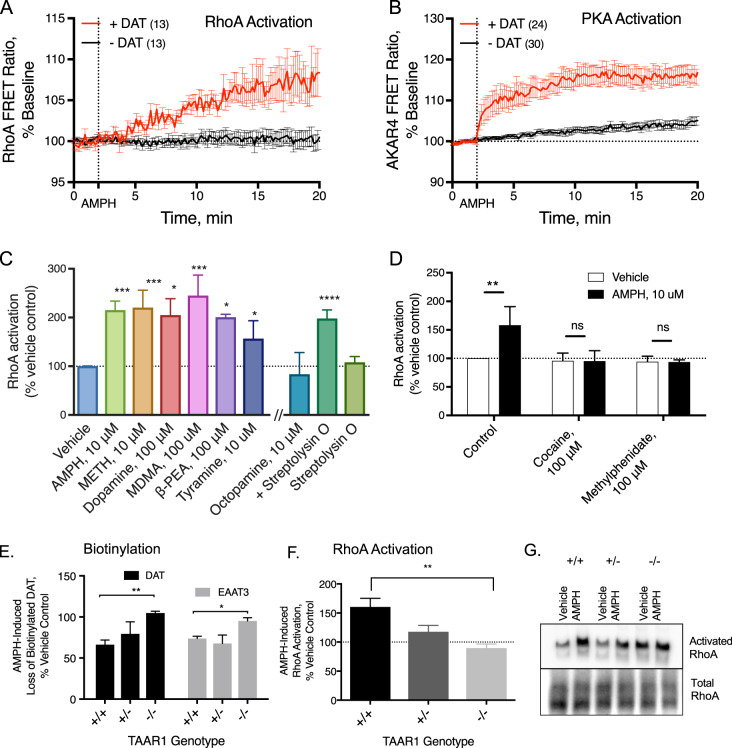
Neurotransmitters, trace amines, and AMPHs activate the small GTPase RhoA in HEK293 cells and midbrain neurons. In HEK293 cells transiently transfected with the DAT and either the FRET sensor for RhoA (**a**) or PKA (**b**) activation, the application of 10 μM AMPH, indicated by the vertical dashed line at 2 min, stimulates activation of these enzymes. However, in cells expressing only the FRET sensor and not the DAT, AMPH does not lead to RhoA or PKA activation (black lines). Activated RhoA was isolated with a GST-isolation assay in HEK293 cells transiently transfected with DAT (**c**). RhoA activation was detected in response to a number of DAT transported TAAR1 agonists. Octopamine did not stimulate RhoA until the cells were permeablized with streptolysin O before octopamine application. The non-transported DAT inhibitors cocaine (100 μM) and methylphenidate (100 μM) did not stimulate RhoA activation on their own and were sufficient to block AMPH-induced RhoA activation (**d**). Biotinylation of cell-surface proteins in acute midbrain slices of wildtype or TAAR1 knockout animals was performed after vehicle or AMPH treatment. AMPH-induced DAT and EAAT3 internalization was abolished in TAAR1 knockout animals (**e**). RhoA activation (**f**, **g**) observed after 15 min of AMPH exposure in acute slices from wild-type mice was absent in the homozygous littermate knockout animals. Heterozygous animals displayed an intermediate level of RhoA activation. Asterisk indicates *p < 0.05, **p < 0.01, ***p < 0.001, and ****p < 0.0001 by one-way ANOVA with Dunnett’s multiple comparisons test for **c**, **e**, **f** and two-way ANOVA with Sidak’s multiple comparisons test for **d**; *n* ≥ 3

Quantification and statistical analysis: Blots were quantified by densitometry with FIJI. Statistical analysis was carried out with Graphpad Prism 6 using one- or two-way ANOVAs with or Dunnett or Sidak corrections. All cell culture assays were performed on a minimum of three different cultures, plated and transfected on at least two separate days. Cell tissue assays were performed on at least three samples on three different days. Where possible, experimenters were blinded to the conditions being tested. All values in the figures indicate mean ± SEM. Precise *N* values that are not indicated in the figure are provided here, reading left to right, as follows: Fig. 1c: 6,5,3,3,6,9,3,3; 1D: 3,3,3,3,3,3; 1E: 4,4,5,3,4,4; 1F: 4,3,4; 2C: 9,9,9,9,9,9; 2D: 9,9,9,9,9,9; 4A: 14,12,11, 12,15,12,12,9; 4B:18,12,18,12,18,12,18,12,18; 4C: 18,18,11,14,12,12,14; 4D: S-26,13–30,Scr-20; 4E: S-29,13–24,Scr-20; 4G: 6,9,9,9; 5A: 5,3,4,3,5,3,4,3.

Drugs and reagents: Primary antibodies used in this study include Rabbit anti-Rho (abcam Ab40673), Rabbit Anti Rho-S188P (abcam Ab41435), Rabbit anti-DAT (Amara Lab generated), Rabbit anti-EAAT3 (Alpha diagnostics EAAC11-A), Chicken anti-Tyrosine Hydroxylase (Aves Labs, Inc. TYH), and Rabbit anti-G_13_ (abcam Ab128900). We used the following secondary antibodies: Donkey anti-chicken Alexa488 (Jackson Immuno Research Labs 120990), Goat anti-rabbit Alexa-568 (Thermo Fisher Scientific A-11036), and Donkey anti-rabbit HRP (Thermo Scientific 31458). Our custom peptides were synthesized by LifeTein. The (+)-Amphetamine hemisulfate [(αS)-α-Methylbenzeneethanamine sulfate] provided by the National Institute on Drug Abuse Drug Supply Program Division of Therapeutics and Medical Consequences. All other drugs used in this study were from Sigma-Aldrich or Tocris.

## Results

### Neurotransmitters, trace amines, and AMPHs enter the cell through the DAT and activate the small GTPase RhoA

Using an enhanced RhoA FRET sensor we [20] first examined whether RhoA could be activated by AMPH in DAT-transfected HEK293 cells, which lack the capacity to synthesize or release biogenic amines (Fig. 1a). We used a RhoA FRET sensor [21] with the replacement of CFP with the third generation fluorophore mCerulean3 [22] to measure activated RhoA signal in response to AMPH (Figs. 1a and S1). As we previously observed in SK-N-SH cells and neurons in culture, AMPH-induced RhoA activation in HEK293 cells was also dependent upon DAT co-expression (Fig. 1a).

AMPH-induced PKA activation was also assessed in the HEK293 cells using the AKAR4 FRET sensor [16] with and without DAT co-expression (Fig. 1b). In cells expressing this FRET sensor we consistently observed a small, slow increase in PKA activation even in the absence of DAT that was also present in vehicle treated controls (data not shown). However, with DAT co-expression, AMPH elicited a dramatic activation in PKA.

To determine whether TAAR receptors mediate AMPH activation of Rho, we examined whether different TAAR1 agonists could enhance RhoA activation. In these assays, we assessed RhoA activation with a pulldown assay (see Materials and methods). As observed with AMPH, the application of methamphetamine (10 μM), DA (100 μM), MDMA (100 μM), β-PEA (100 μM), or tyramine (10 μM) resulted in a robust increase in activated RhoA within 10 min of the addition of drugs (Fig. 1c). The ability of these agonists to activate intracellular TAAR1-mediated RhoA signaling displays a pharmacological profile very similar to that reported for the activation of adenylyl cyclase by surface-expressed TAAR1 [4, 5]. However, it is important to note that the effective concentrations in our system also reflect the kinetics of agonist transport through the DAT, its cytoplasmic stability, as well as its affinity for the receptor.

Notably, the TAAR1 agonist octopamine (10 μM) did not activate RhoA in these cells (Fig. 1c). Octopamine is a potent activator of TAAR1 [4, 5] however, it is a poor substrate for DAT [23]. Because the DAT dependence of the effects of TAAR1 agonists on RhoA activation suggests that TAAR1 agonists act intracellularly to stimulate the receptor, we permeablized the HEK293 cells with streptolysin O to allow octopamine access to the cytoplasm [24]. In the presence of streptolysin O, octopamine produces an approximately twofold increase in activated Rho, whereas streptolysin O had no effect on its own, demonstrating that a TAAR1 agonist that is not a substrate for DAT can activate Rho-dependent signaling once it enters the cell. These studies also imply that entry through the DAT may not be prerequisite for activation of intracellular signaling by TAAR1; in fact, two recently-developed compounds from Roche (RO5166017 and RO5203648) show TAAR1 agonist activity in DAT KO mice and thus may gain access to the cell interior via other avenues [25, 26].

Further evidence supporting the interpretation that DA and AMPH-like compounds act within the cell to produce RhoA activation, we found that the AMPH-induced activation of RhoA was blocked by inhibiting transport through DAT with the non-transported competitive DAT inhibitors cocaine (100 μM) and methylphenidate (100 μM; Fig. 1d). These compounds did not affect RhoA on their own indicating that their actions were through inhibition of AMPH entry into the cell.

### AMPH-stimulated transporter internalization and RhoA activation is not observed in tissue from TAAR1 homozygous knockout mice

The pharmacology of the RhoA activation in HEK293 cells was similar to that of PKA activation by TAAR1. To address whether TAAR1 receptors also mediate RhoA activation in dopamine neurons in the brain and subsequent neurotransmitter transporter trafficking, acute brain slices were prepared from the midbrain of WT and littermate *TAAR1* KO animals and treated with AMPH or vehicle control for 30 min. Assessing biotinylated proteins at the plasma membrane showed that, similar to previous reports [27], AMPH-induced trafficking of DAT was not observed in *TAAR1* KO mice (Fig. 1e). We also found that internalization of EAAT3 was not observed in these animals. Biotinylation data from the heterozygotes was not statistically different from the WT or KO for either the DAT or the EAAT3. In similar studies in acute brain slices, treated with AMPH or vehicle control for 15 min, WT midbrain slices exhibited AMPH-stimulated RhoA activation, when assessed with the GST–RBP pull-down assay, but we found no increase in RhoA activation in AMPH-treated homozygous KO animals (Fig. 1f, g). Heterozygous mice displayed an intermediate phenotype in the RhoA activation assays, though this did not reach statistical significance.

### AMPH does not activate RhoA or PKA in cells that lack the *TAAR1* gene

**Fig. 2:**
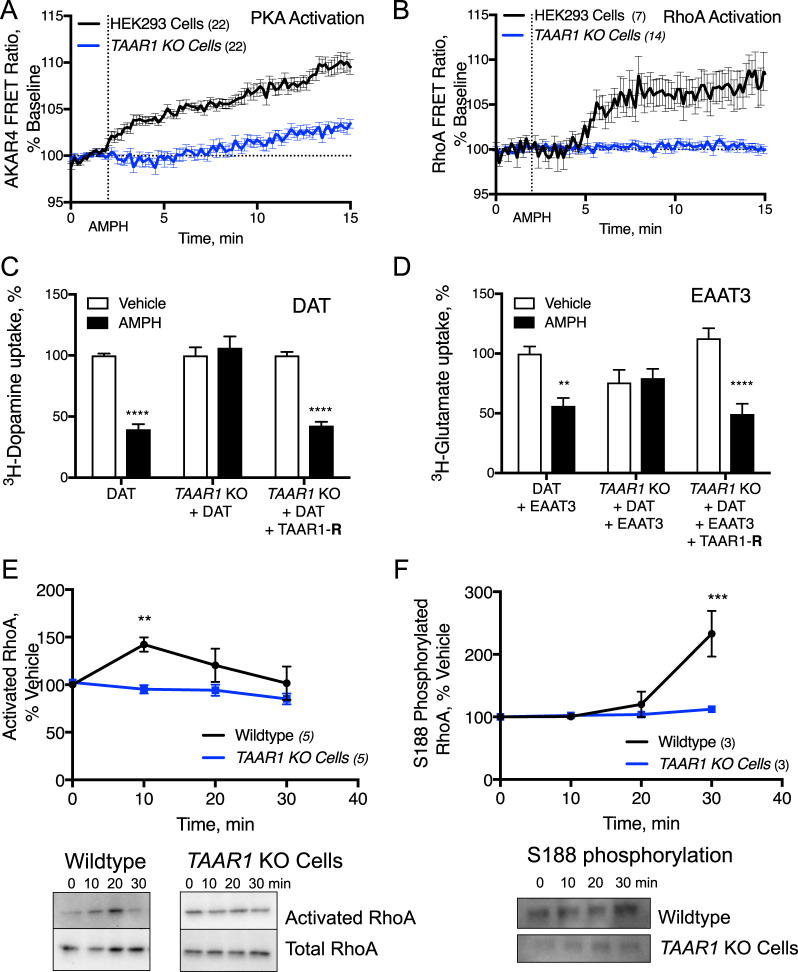
AMPH does not activate RhoA or PKA in cells that lack the *TAAR1* gene. HEK293 cells in which the *TAAR1* gene is intact or knocked out using CRISPR-Cas9 technology (*TAAR1* KO Cells) were transiently transfected with the DAT and AKAR4- or Rho-FRET sensors to measure and the activation of PKA or RhoA, respectively (**a** and **b**). AMPH did not stimulate PKA or RhoA in the *TAAR1* KO line (blue lines), while the responses in their wild-type counterparts (black lines) are similar to those observed in Fig. 1. To assess the effect of the *TAAR1* gene deletion on RhoA-mediated downregulation of DAT and EAAT3, we transiently expressed DAT (**c**) or DAT and EAAT3 in wild-type and *TAAR1* KO cells and (**d**) measured AMPH-induced trafficking of the transporters with ^3^H-neurotransmitter uptake assays. AMPH-induced loss of the activity of both transporters was ameliorated in the knockout cell lines. This effect was rescued by co-transfection with a Cas9 nuclease-resistant TAAR1 construct (TAAR1-R). The time course of RhoA activation was assessed using an affinity-based pulldown assay followed by western blotting (see Materials and methods) in *TAAR1* KO HEK293 cells (blue) and wild-type HEK293 cells (black, **e**). AMPH-induced RhoA activation peaked at 10 min and returned to baseline by 30 min. PKA-mediated phosphorylation of RhoA at S188 (**f**) peaked at the 30-min time point (**p < 0.01, ***p < 0.001, and ****p < 0.0001 by two-way ANOVA with Sidak’s multiple comparisons test; *n* = 9 unless otherwise noted in figure)

To more completely characterize the role of TAAR1 in AMPH-induced PKA and RhoA activation, we designed *TAAR1 knockout cells*. Using CRISPR/Cas-9 technology to disrupt endogenous TAAR1 expression in HEK293 cells (see Materials and methods) we observed that KO of the TAAR1 receptor in HEK293 cells did eliminate activation of PKA by AMPH as assessed by the AKAR4 PKA FRET sensor (Fig. 2a). Similarly, AMPH-mediated RhoA activation was also not evident in the *TAAR1* knockdown cell line (Fig. 2b).

AMPH-stimulated internalization of DAT [2] and EAAT3 [1] are Rho-dependent events, so we assessed whether internalization was also mediated by TAAR1. Using transport activity as a measure of cell surface localization, the uptake of ^3^H-DA or ^3^H-Glu was assessed following AMPH pretreatment of wild-type HEK293 cells and *TAAR1* KO cells transiently expressing either DAT alone or DAT and EAAT3. As observed in studies of *TAAR1* KO mice [27], the AMPH-induced decrease in surface DAT was absent in the *TAAR1* KO cells (Fig. 2c). Furthermore, we found that AMPH-induced loss of EAAT3 function was also absent in the KO cells (Fig. 2d). AMPH-induced internalization of both DAT and EAAT3 was restored in *TAAR1* KO cells transiently transfected with a CRISPR-Cas9 resistant *TAAR1* construct (Fig. 2c, d, see Materials and methods). These data confirm that AMPH-induced cAMP and RhoA signaling is eliminated in our KO cell line, implicating TAAR1 in the initiation of both of these AMPH-activated biochemical cascades.

Cells that lack TAAR1 receptors show no regulation of RhoA by PKA-dependent phosphorylation. We previously showed that increases in PKA activity leads to phosphorylation of RhoA at S188 [2], which removes it from the activated pool [28]. Thus, RhoA activation in response to AMPH rises initially, but then falls with time, as a consequence of S188 phosphorylation [2]. AMPH-treated wild-type HEK293 cells displayed a pattern of RhoA activation that peaked at 10 min (Fig. 2e). RhoA activation returned to baseline by 30 min after AMPH application (Fig. 2e) paralleling the increase in phosphorylation of RhoA (Fig. 2f). However, in cells lacking TAAR1, AMPH treatment produced no changes in RhoA activation. When the phosphorylation state of S188 was assessed, we observed no increase in phosphorylated RhoA after AMPH treatment in the TAAR1 KO cells. These observations confirm that AMPH-mediated PKA activation and phosphorylation of RhoA depends on TAAR1 (Fig. 2f**)** and also support the idea that TAAR1 couples to RhoA and PKA signaling pathways within the same cell.

The role of TAAR1 in AMPH stimulated RhoA activation was confirmed by GST–RBD pull-down assays. We observed a similar pharmacological profile of RhoA activation using either the GST–RBD pull-down or the Rho-FRET sensors. In wild-type HEK293 cells, RhoA activation was stimulated by a number of TAAR1 agonists, but methamphetamine, DA, and β-PEA failed to stimulate RhoA activation in the *TAAR1* KO cells (Fig. S2).

### TAAR1 signaling through PKA and RhoA occur in different intracellular sites

**Fig. 3:**
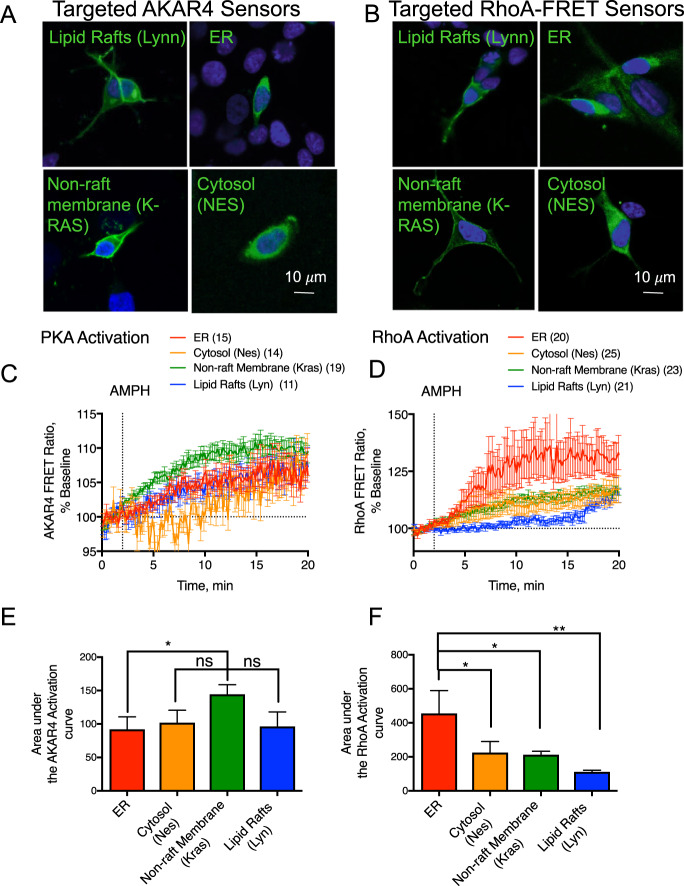
TAAR1 signaling through PKA and RhoA occur in different intracellular sites. SK-N-SH neuroblastoma cell were transfected with DAT and either AKAR4 (**a**) or the Rho-FRET (**b**) sensors to which tags were added to the carboxy or amino termini to dictate localization to various subcellular domains (see Materials and methods). Cellular nuclei were stained with DAPI (blue). PKA activation by AMPH was detected in all compartments (**c**) but favored non-raft membranes. RhoA activation (**d**, **f**) was most robust near the ER (red). (*p < 0.05 and **p < 0.01 by one-way ANOVA with Dunnett’s multiple comparisons test compared with the highest responding compartment; *n*≥10 cells.)

Cumulatively, these data suggest that intracellular TAAR1 activates both PKA and RhoA. To determine whether TAAR1 cAMP- and Rho-signaling pathways are segregated into different subcellular compartments, we used an approach developed by Zhang et al., that fuses compartment-specific targeting motifs to FRET sensors to provide subcellular readouts of signaling pathways. Previous work used this method to target an AKAR4 FRET sensor to the ER, the cytosol, lipid rafts or non-lipid raft plasma membrane domains [16–18, 29]. We fused these same motifs to the RhoA sensor to probe potential subcellular domains where RhoA GTPase cascades might occur. The RhoA sensor was expressed similarly to the various subcellular regions in HEK293 and the neuroblastoma cell line SK-N-SH cells as previously reported for the AKAR4 targeted sensors (Figs. 3a, b, S3A and B).

AMPH-mediated PKA stimulation and signaling through the AKAR4 FRET sensors in the different intracellular compartments was observed in all the subcellular domains in both SK-N-SH (Fig. 3c) and HEK293 cells (Fig. S3C) cells, slightly favoring the non-raft membrane compartment in SK-N-SH cells (Fig. 3e). As a positive control, epinephrine (250 nM) stimulated PKA activation occurred predominantly in lipid-raft plasma membrane compartments in HEK293 cells (Fig. S3E), consistent with previous reports [16, 30].

All of the targeted RhoA sensors responded to the positive control, calpeptin (Fig. S3F) (Schoenwaelder and Burridge, 1999). AMPH-stimulated RhoA activation was particularly pronounced, when the sensor was targeted to the ER in both HEK293 cells (Fig. S3D) and SK-N-SH cells (Fig. 3b, c). The RhoA sensors that were targeted to the cytosol, lipid raft or non-lipid raft membrane domains did show some RhoA activation in response to AMPH, but less than that seen with the ER-targeted sensor. Baseline responses from all of these sensors were stable. These data indicate that TAAR1 receptors signal through RhoA- and PKA-pathways in distinct subcellular compartments; upon activation of TAAR1, RhoA signaling occurs in a discrete pattern near the ER, whereas PKA-signaling extends broadly throughout the cell.

### AMPH-induced RhoA activation in HEK293 cells is mediated by G_13_

The TAAR1 receptor has been demonstrated to couple to the G_s_ G-protein α-subunit, resulting in activation of adenylyl cyclase and elevation of cAMP. However, our data indicate that it also couples to a pathway that facilitates Rho-activation. Several G-protein α-subunits are known to couple to RhoGTPase guanine nucleotide exchange factors that lead to the activation of RhoA including G_q_, G_12_, and G_13_ [31, 32].

**Fig. 4:**
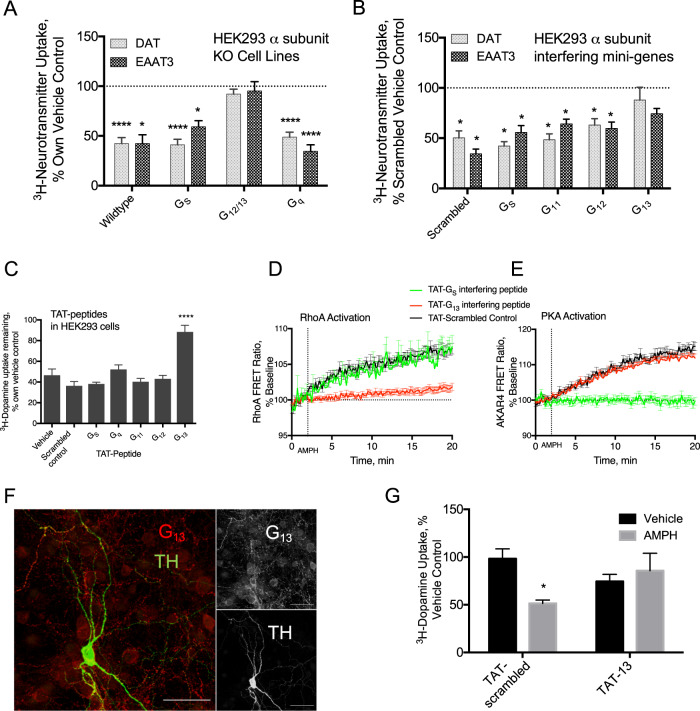
AMPH-induced RhoA activation is mediated by G_13_. HEK293 cells in which various endogenous α-subunits of GPCRs were knocked out were co-transfected with DAT or DAT and EAAT3 (**a**). G_S_ and G_q_ knockout cell lines exhibited similar sensitivity to AMPH as wild-type HEK293 cells with a decreased capacity to transport ^3^H-neurotransmitters following a 30-min pretreatment with AMPH (10 μM). Cells in which G_12/13_ were knocked  out, however, lost sensitivity to AMPH pretreatment in regards to both DAT and EAAT3 trafficking. Wild-type HEK293 cells were co-transfected with DAT or DAT and EAAT3 along with mini-genes that interfere with the function of various α-subunits of GPCRs (**b**; Gilchrist et al. 1999 and 2001). The effect of AMPH on DAT and EAAT3 function were unaltered by the mini-genes that interfere with G_S_, G_11_ or G_12_. However, the effects of AMPH on DAT and EAAT3 were inhibited by co-expression of the G_13_ interfering minigene. We made cell-permeable versions of the alpha-interfering mini-genes by creating peptides of the interfering sequences with the addition of the TAT domain (YGRKKRRQRRR). HEK293 cells that were transiently transfected with DAT were treated with the TAT peptides for 30 min and then with AMPH (10 μM) for 30 min. Similar to the co-expression results, only the G_13_ interfering peptide selectively disrupted AMPH-mediated DAT internalization (**c**). Selectivity of the G_13_ and G_S_ peptides was determined by recording responses of the Rho-FRET (**d**) or the AKAR4-FRET sensor (**e**) in HEK293 cells expressing DAT. AMPH-induced RhoA activation was prevented by pretreatment with the TAT-interfering peptide directed at G_13_ (red), while the G_S_ interfering peptide (green) did not alter RhoA activation (**d**). AMPH-induced PKA activation detected by AKAR4 was blocked by the G_S_ interfering peptide (green), but the G_13_ (red) response was similar to scrambled control (black, **e**). G_13_ expression (red and top panel) was detected in TH(+) cultured neurons (**f**, green and bottom panel). ^3^H-DA uptake in primary midbrain cultures was measured to reflect cell-surface localization of the DAT (**g**). With TAT-scrambled peptides, AMPH pretreatment (30 min, 10 μM) lead to a loss of DAT-mediated ^3^H-dopamine transport capacity but those treated with the TAT-13 interfering peptide, had no sensitivity to the AMPH pretreatment. (Asterisk indicates *p < 0.05, **p < 0.01, *** *p* < 0.001, and **** *p* < 0.0001 by two-way ANOVA with Sidak’s multiple comparisons test compared with vehicle control; *n*≥10 wells per condition)

HEK293 cell lines with selective deletions of the genes for α-subunits G_s_, G_q_, or G_12/13_ [33–35] were transiently transfected with the DAT or the DAT and EAAT3 transporters and treated with AMPH or vehicle control. ^3^H-DA or ^3^H-Glu uptake assays were used to estimate the surface expression of transporters on the plasma membrane. After AMPH treatment, only G_12/13_ KO cells lacked a response to AMPH, consistent with the idea that TAAR1 activation of G_12/13_ α-subunits couples to RhoA signaling in order to mediate AMPH-stimulated DAT and EAAT3 internalization (Fig. 4a). The loss of response in the G_12/13_ KO cells was reversed by co-transfection with the G_13_ α-subunit (Fig. S4).

To further validate the findings obtained using the G_*s*_, G_q_, or G_12/13_ -KO HEK293 lines, we also investigated a model that allows a more transient inhibition of G-protein α-subunits. HEK293 cells were transiently transfected with both the neurotransmitter transporters along with a mini-gene that interferes with G_s_, G_q/11_, G_12_, or G_13_ signaling by expressing a sequence from the C-terminal of the α-subunit that effectively competes for GPCR binding and can selectively prevent α-subunit stimulation [36, 37]. The G_13_ interfering sequence attenuated the effects of AMPH on trafficking of both EAAT3 and DAT (Fig. 4b). However, G_*s*_, G_q/11_, or G_12_ interfering sequences had no effect on trafficking of either transporter.

### Alpha-subunit interfering peptides support the role of G_13_ in AMPH-induced RhoA activation in cell lines and dopamine neurons

With the aim of examining the acute role of G_13_ subunits TAAR1-dependent RhoA activation in neurons, we designed short peptides with the interfering domains of G_s_, G_q_, G_11_, G_12_, or G_13_ [19], conjugated to a cell-permeable TAT peptide (YGRKKRRQRRR). We tested the efficacy of these peptides in HEK93 cells by applying the peptides for 30 min before AMPH treatment, in order for the peptides to penetrate the cell membrane and block α-subunit mediated processes. We found that only the TAT-G_13_ interfering peptide could block AMPH-mediated DAT internalization in HEK293 cells (Fig. 4c).

The specificity of the TAT-G_s_ and TAT-G_13_ interfering peptides for their downstream signaling pathway was tested in HEK293 cells with either the RhoA FRET sensor (Fig. 4d) or the PKA sensor (Fig. 4e). In addition, we wanted to address whether the two signaling pathways, cAMP and Rho, A are linked sequentially or occur in parallel. Do TAAR1 receptors signal through RhoA with cAMP downstream or vice versa or are there two populations of TAAR1 receptors that operate in parallel, as our targeted FRET assays suggest? AMPH-induced PKA activation was blocked by the TAT-G_s_ peptide but not the TAT-G_13_ (Fig. 4e). Similarly, RhoA activation in response to AMPH was blocked by the TAT-G_13_ peptide but not the TAT-G_s_ peptide (Fig. 4d). These data support the efficacy of our TAT interfering-peptides for selectively inhibiting either G_S_ or G_13_ and also refute the idea that the stimulation of PKA and RhoA occurs sequentially. The absence of competition between G_S_ and G_13_ binding also suggests the different membrane environments, protein interactions or some other process restricts the interaction of each of the two populations of TAAR1 receptors to a particular Gα protein subtype.

Endogenous G_13_ expression was examined in primary midbrain cultures. We found that TH(+) neurons (green) were also G_13_(+) (red, Fig. 4f). Immunoreactivity for G_13_ was observed in discrete, cytoplasmic puncta consistent with a localization at or near ER membranes. We also attempted to examine the intracellular localization of TAAR1 by immunolabeling or by expressing a tagged-TAAR1 protein. Unfortunately, the expression of TAAR1 is below the level of detection by the commercial antibodies currently available. We have also observed that overexpression of TAAR1 or the epitope-tagged TAAR1 favors G_S_ coupling and occludes G_13_-dependent RhoA activation through the endogenous receptor (data not shown). Thus in our studies, we avoided overexpression systems and used targeted FRET sensors to characterize *endogenous* TAAR1 signaling in intracellular compartments.

**Fig. 5:**
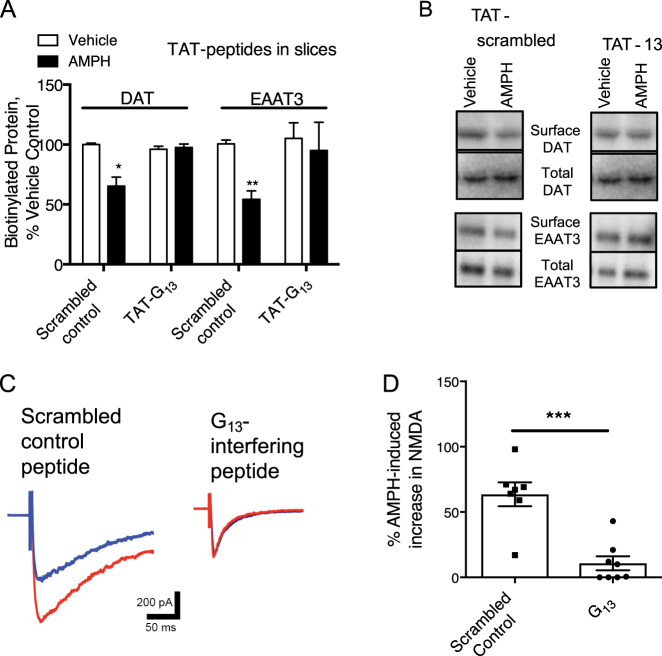
G_13_ mediates AMPH-induced internalization of DAT and EAAT3, as well as AMPH-induced potentiation of glutamatergic responses. In acute brain slices, cell-surface expression of DAT and EAAT3 were assessed by biotinylation assays (**a** and **b**). Pretreatment with the G_13_ interfering peptide blocks AMPH-mediated internalization of both of the neurotransmitter transporters. The G_13_ inhibiting peptide also blocks AMPH-mediated potentiation of NMDA synaptic currents in mouse midbrain dopamine neurons (C and D). Example traces of evoked NMDA synaptic currents in Mg^2+^-free extracellular solution. Left, in the presence of an intracellular scrambled version of the peptide (10 µM) AMPH (red) potentiates the baseline synaptic current (blue), similar to previous observations. Right, AMPH (red) has no effect on the baseline current (blue) in the presence of the G_13_ inhibitor peptide applied intracellularly (10 µM). Compiled data showing a lack of significant potentiation by AMPH in the cells recorded with the G_13_ inhibitor peptide (**d**). Cartoon of AMPH-mediated signaling through two populations of TAAR1 (*t*_(13)_ = 5.16, *p* = 0.0002; *p* < 0.001 by paired *t*-test; *p < 0.05 **p < 0.01 by two-way ANOVA with Sidak’s multiple comparisons test for panels A and E; *n*≥3)

In primary midbrain cultures, DAT-mediated ^3^H-DA uptake can be used as an assay for the presence of the endogenous DAT at the cell surface. We found that the effects of pretreatment with AMPH that typically induces downregulation of DAT in primary cultures (10 μM, 30 min), were blocked by the TAT-G_13_ interfering peptide (Fig. 4g). We also examined the effect of the G_13_ interfering peptide on mature mouse brains expressing TAAR1 and their impact upon the downstream effect of DAT and EAAT3 internalization. When acute brain slices were pretreated with the G_13_-interfering TAT peptide, the AMPH-mediated loss of cell surface, biotinylated DAT, and EAAT3 was blocked (Fig. 5a, b). AMPH-mediated potentiation of glutamatergic signaling to midbrain dopamine neurons is mediated by internalization [38] of EAAT3 [1, 39]. Consistent with its role, TAAR1–G_13_–RhoA in this pathway is provided by evidence that the G_13_ inhibitory peptide blocks potentiation of NMDA-mediated currents in midbrain dopamine neurons (Fig. 5c, d).

## Discussion

**Fig. 6:**
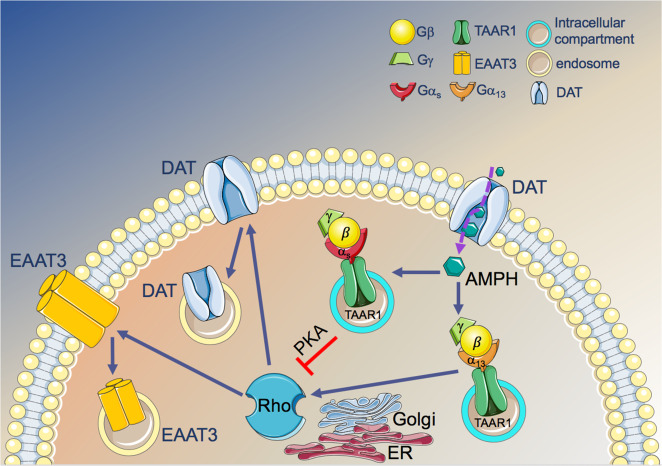
AMPH enters the cells through the DAT found at the plasma membrane. Once inside the cell, AMPH binds to G13-coupled TAAR1 receptors that stimulate RhoA activation near the ER. RhoA mediates endocytosis of the neurotransporters DAT and EAAT3. AMPH also stimulates GS-coupled TAAR1 receptors that propagate PKA signaling throughout the neuron. Downstream PKA activation leads to phosphorylation of the RhoA and stops transporter internalization. (Created using SMART server medical art.)

These studies strongly implicate the TAAR1 as the intracellular target of AMPH that initiates the trafficking effects of AMPH on DAT and EAAT3 (Fig. 6). We report here that the cytoplasmic activation of the TAAR1 receptor by intracellular AMPHs leads to activation of both PKA and RhoA signaling pathways. PKA activation occurs through coupling of the receptor to G_S_-subunits resulting in a signal that propagates in intracellular compartments throughout the cell, whereas RhoA activation occurs through TAAR1 coupling to G_13_ subunits more preferentially localized near the ER. Using TAT peptides that interfere with the coupling of specific G-proteins as well as targeted RhoA FRET sensors, we have identified a population of TAAR1 receptors that couple to G_S_ and stimulate PKA throughout the cell in response to several agonists, including AMPH. These same agonists also stimulate a distinct population of TAAR1 receptors that couple through G_13_ to activate RhoA signaling at or near the ER.

### AMPH signaling in dopamine neurons is mediated by an intracellular GPCR

Traditionally, GPCR signaling initiates with binding of an extracellular ligand at the cell surface followed by activation of a series of biochemical events within the cell. However, recent studies have demonstrated that GPCRs stimulated at the cell membrane and internalized through a beta-arrestin-mediated process can continue signaling from endosomal compartments, facilitating signaling to intracellular regions distant from the plasma membrane (for review see [40]). Our data, however, indicate that TAAR1 appears to reside and signal exclusively from one or more intracellular compartments, because activation of the receptor by agonists depends on expression of a membrane transporter or permeabilization of the cell membrane (Fig. 1).

By examining the signaling of endogenous TAAR1 in HEK293 cells and SK-N-SH cells, we found that agonists of TAAR1 must reach the cytoplasm in order to initiate signaling either through transport by a plasma membrane transporter (Fig. 1a, b**)** or streptolysin O generated perforations in the plasma membrane (Fig. 1c) to gain access to the receptor localized inside the cell. Downstream effects of AMPH activation of TAAR1 in neurons, including DAT and EAAT3 internalization and subsequent enhanced glutamatergic signaling, are all dependent upon DAT expression for AMPH entry into the cell [1, 2, 39]. Others have shown that direct intracellular application of AMPH through an electrophysiological recording pipette triggers DAT internalization in the absence of a functional DAT [3] further supporting a role for intracellular TAAR1 signaling as a prerequisite for AMPH-dependent trafficking events.

### Orientation of the TAAR1 receptor

TAAR1 is a class A GPCR that belongs to the Rhodopsin family [41]. These proteins have seven-transmembrane helices that span throughout the cell membrane, an extracellular amino-terminus (N-terminus) and a cytoplasmic C-terminus. The ligand binds in the cavity formed within the transmembrane bundle that is exposed to the extracellular environment, while G-protein binding is generally ascribed to several intracellular loops and the C-terminus and occurs in the inner leaflet of the plasma membrane [42]. We and others have observed TAAR1-dependent PKA signaling in overexpression systems in the absence of DAT, suggesting that surface applied agonist can access the ligand binding domain and activate the small proportion of receptors that make it to the cell surface. Thus, it appears likely that TAAR1 orientation maintains the C-terminus in the cytoplasm in order to enable G_S_ and G_13_ signaling. However, this topological arrangement would orient the ligand-binding domain of the receptor within the lumen of the intracellular membrane compartment in which it is found. These data suggest that agonists would need to permeate the cell membrane as well as the membrane of an intracellular compartment in order to reach the ligand binding domain. Most amphetamines and a number of other TAAR1 agonists require a transporter to cross the plasma membrane. However intracellular compartments have different lipid composition and geometry that may allow passage of the agonists across the membrane in order to reach the luminal ligand binding site. In fact, tyramine and β-PEA have been shown to readily diffuse across synthetic lipid bilayers [43] and the relatively high lipophilicity of amphetamines may also be a factor [44]. The TAAR1 agonists from Roche (RO5166017 and RO5203648) also display effects that are independent of DAT expression supporting a mechanism of entry into the cells distinct from the plasma membrane transporter [25, 26]. Access to the ligand binding site from the cytoplasm could also be achieved through a variety of other mechanisms. Structural studies have demonstrated that some classes of GPCRs have ligand-binding domains that are accessible to ligands from both inside and outside the cell [45]. Alternatively, there could be yet another membrane transporter that facilitates access of TAAR1 ligands to the lumenal face of the compartment where TAAR1 resides. A mechanism involving glutamate transport by a glutamate transporter localized on ER membranes has been proposed to explain observations of intracellular mGluR5 signaling from the ER and nucleus [46]. In fact, the recently described targeting of the organic cation transporter, OCT3 to the outer nuclear membrane could facilitate ligand transport and receptor activation in that compartment [47]. The ability of an endogenous trace amine, tyramine, to be transported by OCT2 suggests another alternative [48].

### Two spatially-distinct signaling pathways are linked to TAAR1 activation

Our data suggest that there are distinct populations of TAAR1 receptors that differ in their coupling to G_S_ or G_13_ G-protein subunits. The observation that the G_S_ or G_13_ interfering peptides, which interact with the same domains on TAAR1, display effects that are highly-selective for their respective pathways (Fig. 4d, e) implies a remarkable precision in the localization and organization of the two TAAR1 signaling pathways. It remains unclear whether the two receptor populations are differentially localized or whether they colocalize and are linked to pathways that propagate through different compartments

Specificity of TAAR1 coupling to G_S_ or G_13_ may be facilitated through a number of different mechanisms. Phosphorylation or any number of other post-translational modifications of a subset-population of TAAR1 receptors may increase their affinity for one over the other. Protein–protein or lipid–protein interactions may also dictate conformations that favor coupling to distinct G-proteins, as described recently for PIP2 and class A GPCRs [49]. Receptor-G-protein precoupling, where G-proteins exist in a complex with GPCRs prior to receptor activation, has been proposed as a mechanism to account for some of the specificity of receptor-G protein coupling, as well as for the rapid kinetics of intracellular responses [50].

Our results using the TAT-interfering peptides argue against the notion that the two pathways operate sequentially, as hypothesized for GPCRs that change affinity for Gα subunits after stimulation (see Friedman et al. 2002; Chen-Izu et al. 2000; Hill et al. 2003 for reviews). If RhoA-activation preceded PKA activation, the TAT-G_13_ interfering peptide would also have inhibited subsequent G_S_ signaling and PKA activation that our FRET sensor assay did not detect (Fig. 4e). Similarly, the TAT-G_S_ interfering peptide did not inhibit AMPH-induced RhoA activation, indicating that G_13_ coupling does not precede G_S_ coupling (Fig. 4d).

Overall, our experimental data support the interpretation of two spatially distinct pathways of TAAR1 signaling. The interfering mini-gene peptides we used are comprised of sequences from the C-terminus of the α-subunit of each G-protein. Despite the differences in the sequence of the C-terminus between the G_S_ and G_13_ subunits that give the mini-genes their specificity, they are likely to form contacts within the same binding pocket of the TAAR1 receptor. This suggests that a single population of TAAR1 receptors activated by both G_S_ and G_13_ could not mediate the distinct signaling cascades we observe because both G signaling pathways would be inhibited simultaneously once either of the mini-gene peptides binds to the TAAR1 binding site. In fact, our data illustrate the opposite effect: each mini-gene peptide inhibits signaling to its cognate G protein subunit with precise specificity.

It is important to note that both FRET sensors assess signaling events that occur downstream of TAAR1 activation and thus the resolution of the subcellular domains, where TAAR1 signaling occurs may reflect later steps in the pathway. AKAR4 is a PKA target that is linked to TAAR1 activation by a diffusible second messenger and it is difficult to know whether the broad localization observed with the AKAR4 sensor correlates with the distribution of TAAR1-G_S_ or whether it simply reflects the compartmentalization of downstream signaling events. RhoA activation measured by the RhoA-FRET sensor is also linked to TAAR1 activation by a diffusible factor, perhaps PDZ RhoGEF, which has been shown to regulate G_13_ interactions in brain (see Momotani et al. 2012 and Siehler 2009 for reviews); however, the Rho-FRET sensor produced a more pronounced activation when localized to the ER supporting distinct subcellular domains of activity for each of these pathways.

### Role of AMPH-stimulated activation of RhoA and PKA and DAT/EAAT3 internalization

Although the precise physiological role of TAAR1/G_13_-mediated DAT internalization remains to be established, it is likely that this pathway evolved to regulate the actions of endogenous amines including β-PEA, tyramine, 3-methoxytyramine, and DA. DA is a TAAR1 agonist (Fig. 1c) and recently, it was shown that the sustained elevation of extracellular DA seen following burst firing appears to be a consequence of DAT internalization by a Rho-dependent mechanism [51]. These results provide the first evidence that dopamine itself can act as a TAAR1 agonist to trigger transporter internalization in vivo.

TAAR1 signaling also has the potential to serve as a sensor of cytoplasmic DA and its metabolites. At high cytosolic concentrations, DA becomes neurotoxic due to the formation of reactive oxygen species and quinones that can cause damage to the neuron [52]. Under normal conditions, DA transported through the DAT from the extracellular space is rapidly repackaged into synaptic vesicles or enzymatically metabolized. However, if cytoplasmic DA concentrations saturate these mechanisms, the reduction in surface levels of DAT mediated by TAAR1/G_13_ signaling may provide an additional means for maintaining free cytosolic DA below toxic levels.

MDMA and DA have been defined as “partial agonists” for TAAR1 receptors expressed at the cell surface that signal through PKA; however, the pharmacological profile of the two compounds for G_13_-coupled RhoA activation has not yet been fully characterized. A detailed kinetic analysis of the ability of the various compounds to activate intracellular TAAR1 and whether they display preferential signaling though one of the two pathways is a critical goal for future studies.

The role of TAAR1 in the actions of psychostimulants may be attributed to its direct affinity to these ligands but is also affected by the downstream signaling which it stimulates. This study identifies the spatial distinction of two classes of TAAR1 receptors coupled to either G_S_ and PKA activation or G_13_ and RhoA activation and is a critical component of how these drugs act. This model suggests new avenues of exploration for the study of GPCRs, as well as the actions of monoaminergic substances in the brain.

## Supplementary information

Supplementary Figures

Supplemental Figure Legends
