# Supplementary figures and images for: Amphetamines signal through intracellular TAAR1 receptors coupled to Gα13 and GαS in discrete subcellular domains
Source: Mol Psychiatry. 2019 Aug 9;26(4):1208–23. doi: 10.1038/s41380-019-0469-2 (PMC7038576; doi:10.1038/s41380-019-0469-2)

Figure S1

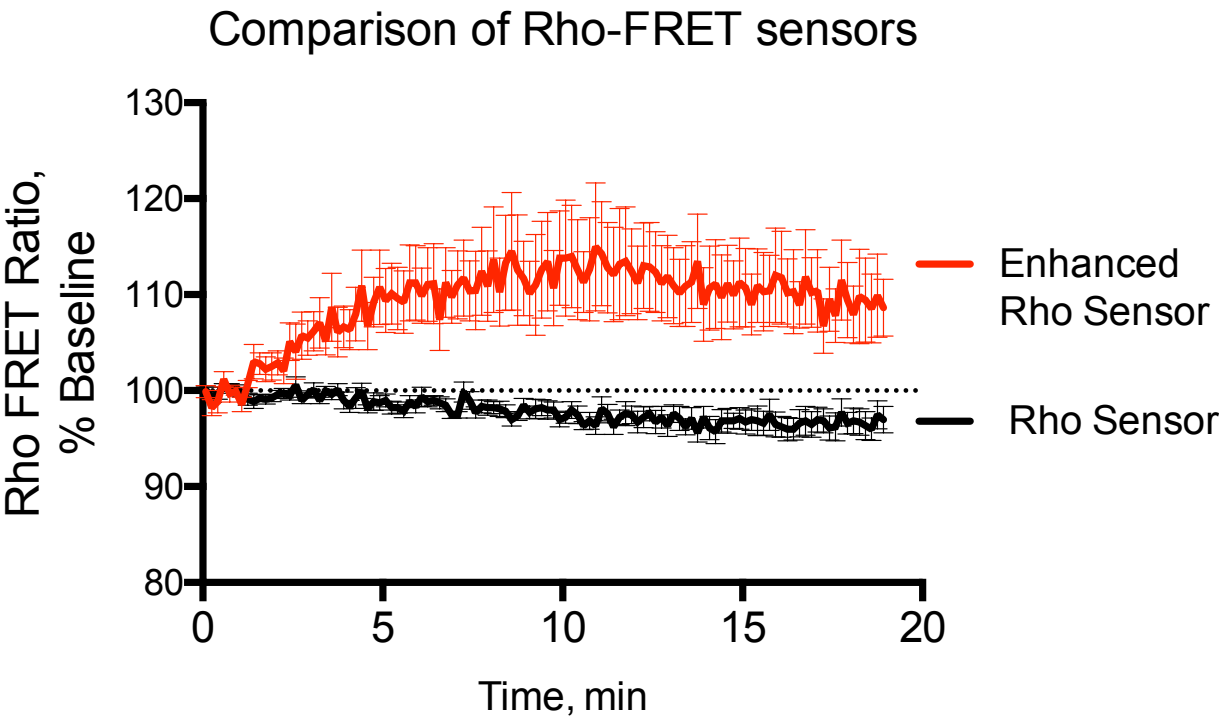

Figure S2

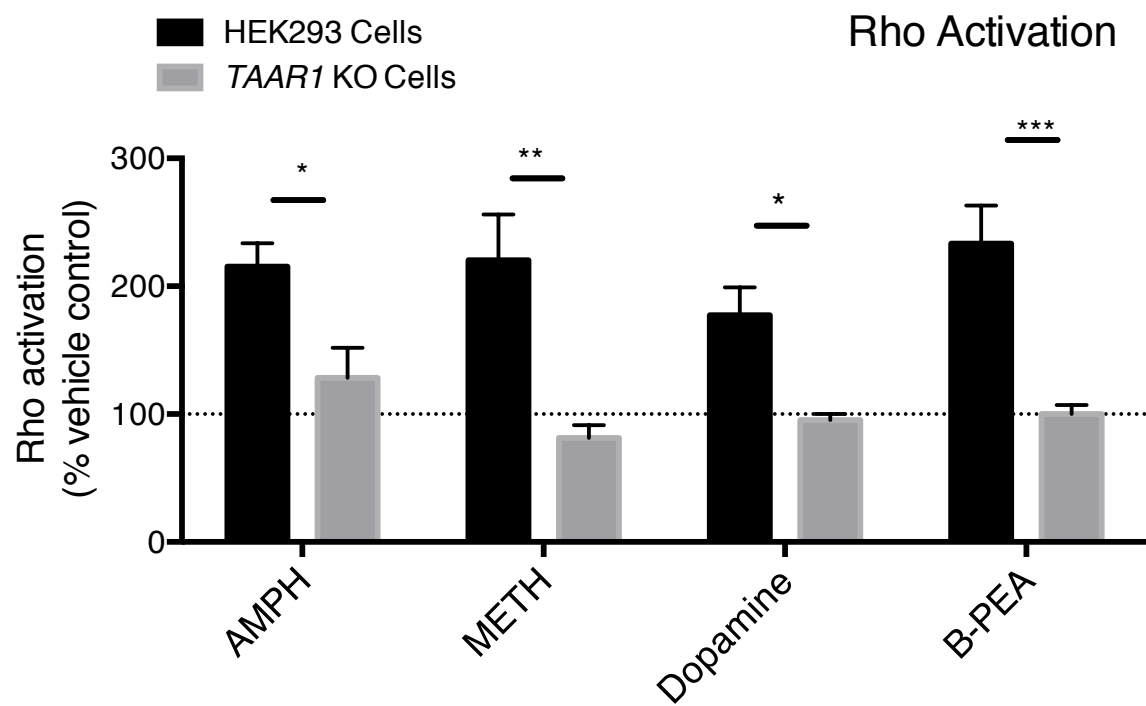

Figure S3

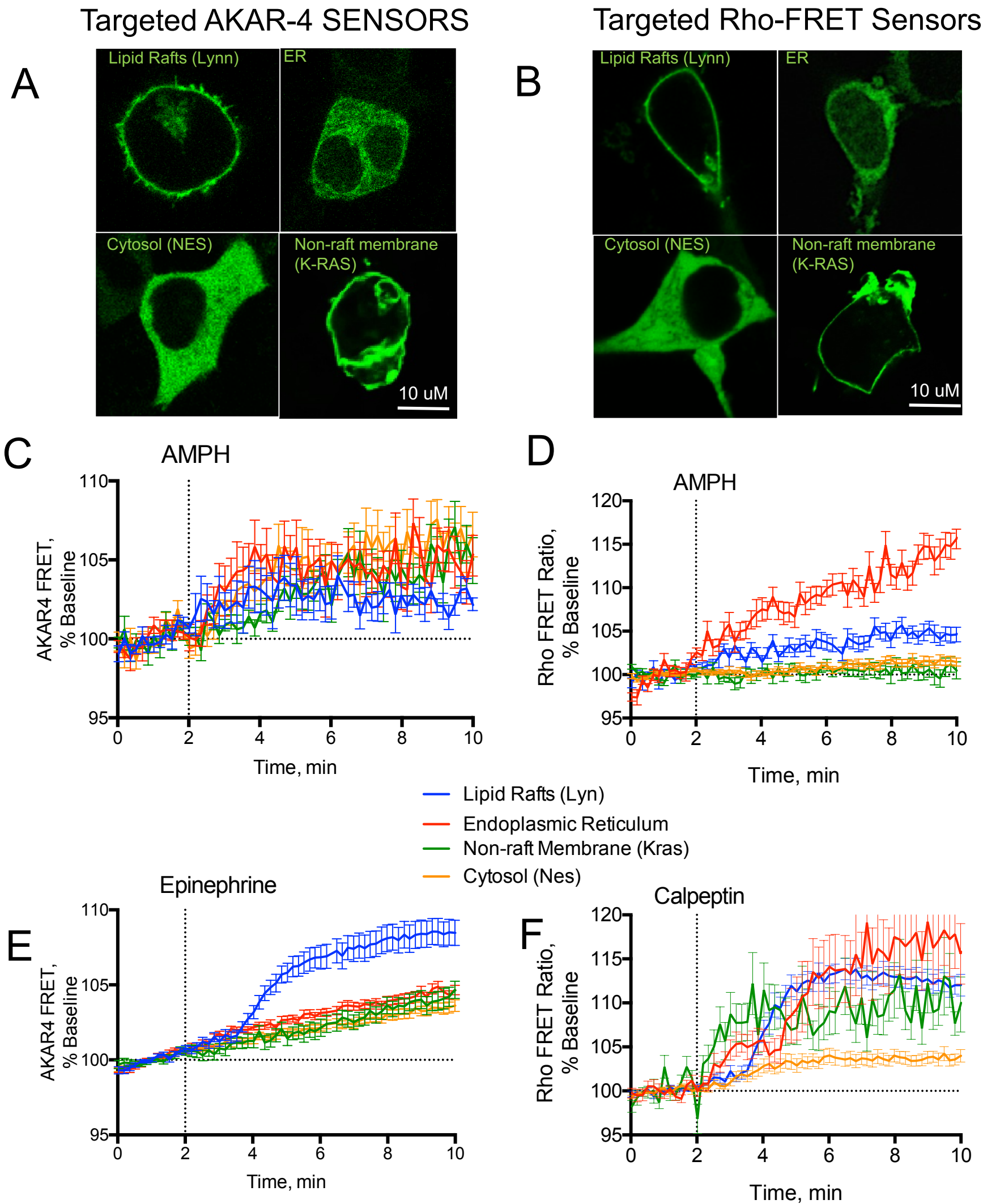

Figure S4

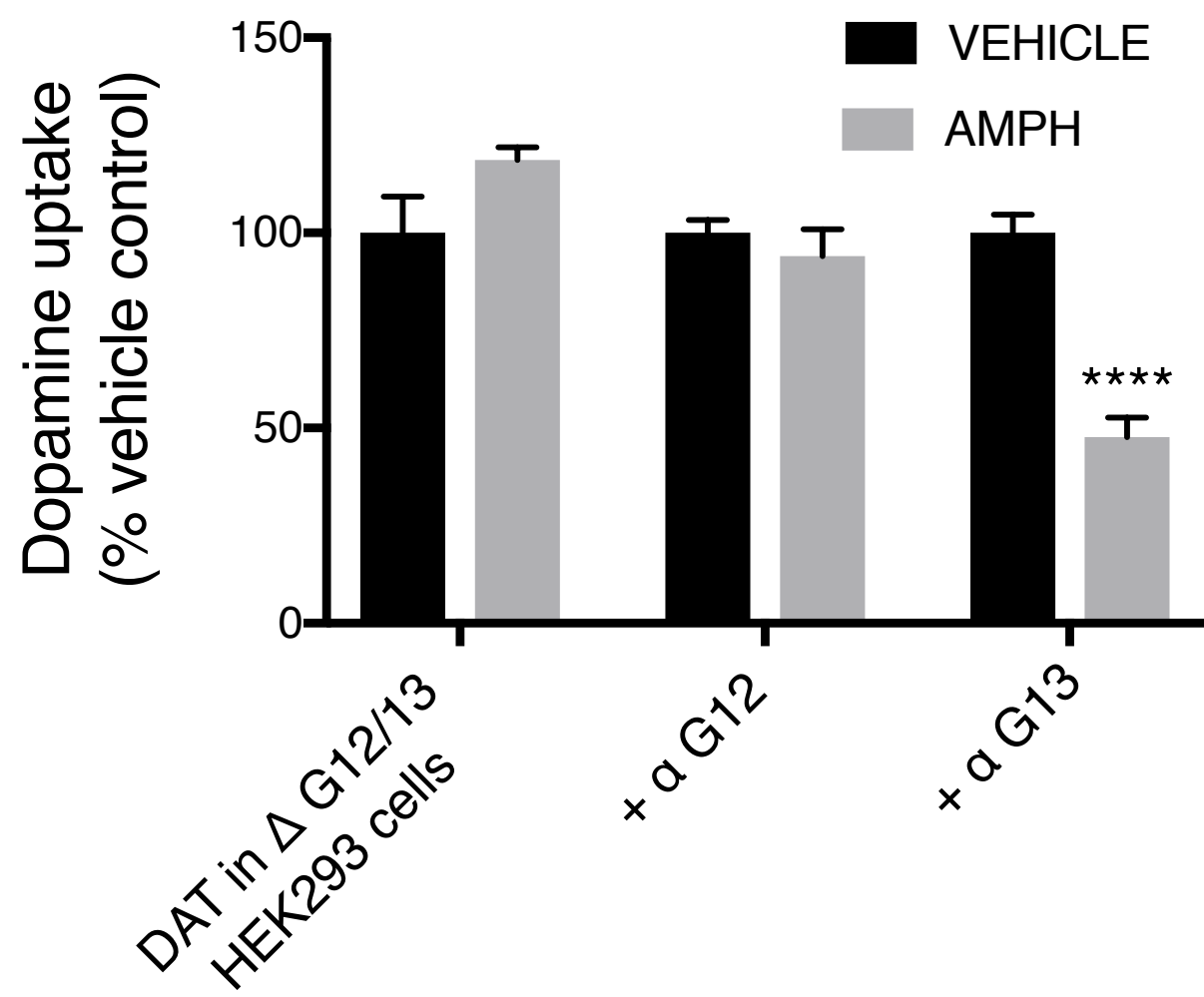

Supplement: Supplementary file 1 — Supplementary Figures [file 41380_2019_469_MOESM1_ESM.pdf]
