# Supplementary material for: Amphetamines signal through intracellular TAAR1 receptors coupled to Gα13 and GαS in discrete subcellular domains
Source: Mol Psychiatry. 2019 Aug 9;26(4):1208–23. doi: 10.1038/s41380-019-0469-2 (PMC7038576; doi:10.1038/s41380-019-0469-2)
Supplement: Supplementary file 2 — Supplemental Figure Legends [file 41380_2019_469_MOESM2_ESM.docx]

**Supplemental Figure Legends**

**Supplementary Figure 1**. **FRET-sensors of RhoA Activation in HEK293 cells.** The first RhoA sensor that we tried did not detect any RhoA activation in DAT-transfected HEK293 cells in response to AMPH, 10 μM (black line). Our enhanced sensor, however, was able to detect AMPH-induced RhoA activation (red line).

**Supplementary Figure 2**. **Pharmacology of RhoA-activation in *TAAR1 KO* cells.**  Naïve and *TAAR1 KO* HEK293 cells were transiently transfected with DAT, exposed to drug treatments (10 μM) for ten minutes, and RhoA activation was assessed by the GST-affinity pull-down assay. RhoA activation was detected in wildtype HEK293 cells in response to AMPH, METH, dopamine and β-PEA however, *TAAR1 KO* cells were unresponsive to all drug treatments. (* p<0.05, ** <0.01 and ***<0.001 by two-way ANOVA with Sidak’s multiple comparisons test; n≥4).

**Supplementary Figure 3. AMPH-induced PKA and RhoA responses in HEK293 cells occur in distinct subcellular compartments.** HEK293 cells were transiently transfected with AKAR4 (**A**) or Rho-FRET (**B**) sensors directed by targeting motifs to various subcellular compartments (green). AKAR4 FRET responses to AMPH were found throughout the cells (**C**), while all compartments did exhibit responses to epinephrine (**E**), consistent with previously published observations. AMPH-induced RhoA activation (**D**) was most dramatic in the ER. All of the targeted RhoA sensors did response to calpeptin (**F**).

**Supplementary Figure 4. Rescue of G12/13 knockout HEK293 cells.** HEK293 cells lacking the G12 and G13 subunits were transiently transfected with DAT or DAT and the G12 subunit or DAT and the G13 subunit. Pretreatment of these cells with AMPH, 10 uM, had no effect on the DAT transport capacity, unlike wildtype HEK293 cells (see **Figure** **4A**). Addition of the G12 subunit was not sufficient to rescue the effect. Co-transfection of the G13 subunit, however, was sufficient to recover AMPH-mediated DAT internalization. (****<0.0001 by two-way ANOVA with Sidak’s multiple comparisons test; n≥4).
